# *Candida* Administration in 5/6 Nephrectomized Mice Enhanced Fibrosis in Internal Organs: An Impact of Lipopolysaccharide and (1→3)-β-D-Glucan from Leaky Gut

**DOI:** 10.3390/ijms232415987

**Published:** 2022-12-15

**Authors:** Somkanya Tungsanga, Kanyarat Udompornpitak, Jesadakorn Worasilchai, Tharit Ratana-aneckchai, Dhammika Leshan Wannigama, Pisut Katavetin, Asada Leelahavanichkul

**Affiliations:** 1 Division of Nephrology, Department of Medicine, Faculty of Medicine, Chulalongkorn University, Bangkok 10330, Thailand; 2 Division of General Internal Medicine-Nephrology, Department of Medicine, Faculty of Medicine, Chulalongkorn University, Bangkok 10330, Thailand; 3 Department of Microbiology, Faculty of Medicine, Chulalongkorn University, Bangkok 10330, Thailand; 4 Antimicrobial Resistance and Stewardship Research Unit, Department of Microbiology, Faculty of Medicine, Chulalongkorn University, Bangkok 10330, Thailand; 5 School of Medicine, Faculty of Health and Medical Sciences, The University of Western Australia, Nedlands 6009, Australia; 6 Center of Excellence in Translational Research in Inflammation and Immunology (CETRII), Faculty of Medicine, Chulalongkorn University, Bangkok 10330, Thailand

**Keywords:** fibrosis, *Candida*, gut-derived uremic toxins, gut leakage, 5/6 nephrectomized mice, chronic kidney disease

## Abstract

Uremic toxins and gut dysbiosis in advanced chronic kidney disease (CKD) can induce gut leakage, causing the translocation of gut microbial molecules into the systemic circulation. Lipopolysaccharide (LPS) and (1→3)-β-D-glucan (BG) are the major gut microbial molecules of Gram-negative bacteria and fungi, respectively, and can induce inflammation in several organs. Here, the fibrosis in the kidney, liver, and heart was investigated in oral *C. albicans*-administered 5/6 nephrectomized (*Candida*-5/6 Nx) mice. At 20 weeks post 5/6 Nx, *Candida*-5/6 Nx mice demonstrated increased 24 h proteinuria, liver enzymes, and serum cytokines (TNF-α, IL-6, and IL-10), but not weight loss, systolic blood pressure, hematocrit, serum creatinine, or gut-derived uremic toxins (TMAO and indoxyl sulfate), compared to in 5/6 Nx alone. The gut leakage in *Candida*-5/6 Nx was more severe, as indicated by FITC-dextran assay, endotoxemia, and serum BG. The areas of fibrosis from histopathology, along with the upregulated gene expression of Toll-like receptor 4 (*TLR-4*) and *Dectin-1*, the receptors for LPS and BG, respectively, were higher in the kidney, liver, and heart. In vitro, LPS combined with BG increased the supernatant IL-6 and TNF-α, upregulated the genes of pro-inflammation and pro-fibrotic processes, *Dectin-1*, and *TLR-4* in renal tubular (HK-2) cells and hepatocytes (HepG2), when compared with LPS or BG alone. This supported the pro-inflammation-induced fibrosis and the possible LPS–BG additive effects on kidney and liver fibrosis. In conclusion, uremia-induced leaky gut causes the translocation of gut LPS and BG into circulation, which activates the pro-inflammatory and pro-fibrotic pathways, causing internal organ fibrosis. Our results support the crosstalk among several organs in CKD through a leaky gut.

## 1. Introduction

Chronic kidney disease (CKD) has been acknowledged as a significant global burden [1], leading to an accumulation of different metabolic substances known as “uremic toxins”. Such toxins, which are primarily produced by food components or metabolic processes in the body, can lead to several consequences, including CKD progression, cardiovascular complications, and pulmonary disorders [2]. A few circulating uremic toxins, including trimethylamine N-oxide (TMAO), indoxyl sulfate, p-cresol sulfate, hippuric acid, and phenylacetic acid, are produced in the gastrointestinal GI tract, and are referred to as gut-derived uremic toxins [3]. The accumulated toxins in advanced CKD are compensatorily excreted into the intestinal system, which promotes the proliferation of pathologic bacteria, referred to as “gut dysbiosis” [4]. During gut dysbiosis, there is an increased production of gut-derived uremic toxins, and the toxins (both gut and non-gut derivatives) might damage intestinal epithelial tight junctions, which causes the so-called “gut leakage” or “leaky gut” or “gut translocation”—translocation of microbial molecules from the gut into the bloodstream [5]. Although gut-derived uremic toxins originate in the intestine, they can spread throughout the body and cause damage to various tissues, including the intestines, heart, liver, and kidneys [6,7,8]. The term “gut–kidney axis” refers to the vicious loop in which uremic toxin buildup and gut dysbiosis intensify as CKD progresses [9]. Particularly, when the microbial compounds, especially lipopolysaccharide (LPS; a major molecule on the cell wall of Gram-negative bacteria) and (1→3)-β-D-glucan (BG; the main component of the *Candida* cell wall), from the intestinal translocation activate their receptors, it leads to the enhanced inflammatory responses that further facilitate the progression of CKD [5]. Notably, Gram-negative bacteria and *Candida albicans* are the microbes with the most and the second most abundance in the human intestine. Therefore, increases in both molecules through a leaky gut might lead to clinical adverse effects [10].

However, fungi are less prominent in the mouse gut than in the human intestine, and the influence of intestinal fungi in mouse models is possibly underestimated [11]. Contrary to what has been found in human feces [12], mouse feces do not have enough *Candida* spp. to be detectable in stool cultures [11]. Although the presence of *Candida* spp. in the gut does not directly cause disease, gut fungi alter the gut microbiota and provide a higher BG in gut content [13], which possibly worsens systemic inflammation following a gut barrier malfunction (gut leakage) through the systemic immune responses against BG [14,15,16,17,18,19,20]. Indeed, the possible fungal mechanisms that interfere with the growth of specific bacteria (mostly the lower virulence) in the gut are mentioned, including some bactericidal molecules (yeast killer toxins, *Candida* exotoxin, and endogenous alcohol) [21,22,23,24], and the competition for certain nutrients [25]. Meanwhile, *Candida* spp. possibly facilitate the growth of specific bacteria with the glucanase enzyme, which can digest BG from the fungal cell wall [26]. Despite the possibility of both symbiotic and antagonistic impacts of *Candida* on gut bacteria, gut fungi alter microbial homeostasis, and the oral administration of *Candida* causes gut dysbiosis, with leaky gut and inflammatory reactions observed in several mouse models [27,28,29,30]. Indeed, explorations into the impacts of gut fungi through oral administration in mice are possible due to the naturally lower abundance of fungi in the mouse gut. Indeed, the increase in fecal fungi after oral administration in mice is reported [31,32].

Gut translocation of LPS and BG causes systemic inflammation [10], and chronic systemic inflammation possibly facilitates fibrosis in several organs as increased serum BG in chronic alcoholism (humans and mice) enhances liver fibrosis, as previously demonstrated [33]. Interestingly, the activation of Dectin-1, a recognition receptor of BG, induces fibrosis in several organs (heart, liver, and lung) [34,35,36,37]. For kidney fibrosis, there are only a few investigations on the impact of gut fungi and BG on CKD. As such, our previous publication demonstrates no difference in renal fibrosis between in CKD mice with or without *Candida* gavage at 16 weeks after 5/6 nephrectomy (5/6 Nx), despite a synergistic pro-inflammatory effect of LPS plus BG [18]. However, the pro-fibrotic impact of LPS and BG on other organs, and the influence on the kidney after extended observation, might be different. Due to (i) the worsening renal fibrosis and CKD progression by systemic inflammation [38] and endotoxemia [39,40], (ii) the induction of gut leakage by systemic inflammation and uremia [5] with the attenuation of leaky gut by probiotics [28,29,30,31,32,33,34,35,36,37,38,39,40,41,42], and (iii) the association among kidney and other internal organs (gut–liver–kidney and gut–heart axis) [10,43], the presence of fungi in the gut with uremia-induced leaky gut might worsen fibrosis in several organs.

Here, 5/6 Nx and *Candida* gavage were observed for 20 weeks with the exploration of fibrosis in other internal organs (liver and heart) along with in vitro experiments. To understand the pathophysiologic effects of fungi on fibrosis, BG and LPS (the main cell wall components of fungi and Gram-negative bacteria, respectively) were tested on hepatocytes (HepG2 cells) and renal tubular cells (HK2 cells).

## 2. Results

### 2.1. The Presence of Candida in the Uremic Gut Enhanced Injury in Internal Organs (Kidney, Liver, and Heart) through Systemic Inflammation

**Figure 1:**
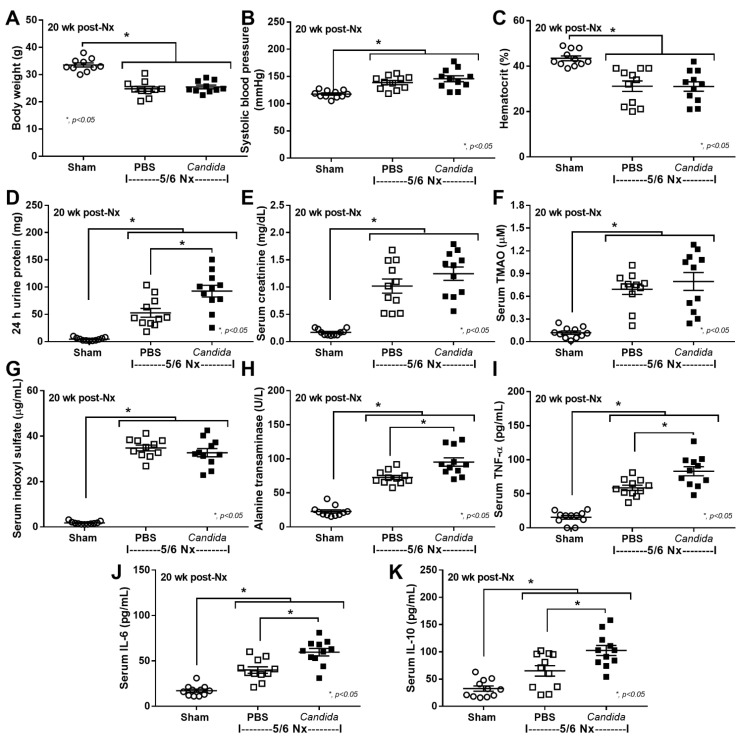
The characteristics of Sham control and 5/6 nephrectomized (5/6 Nx) mice with phosphate buffer solution (PBS) or *Candida* administration (*Candida*) at 20 weeks (wk) after right nephrectomy (20 week post Nx) (see method), as indicated by body weight (**A**), mean arterial pressure (**B**), hematocrit (**C**), renal function (24 h urine protein and serum creatinine) (**D**,**E**), serum gut-derived uremic toxins, including trimethylamine N-oxide (TMAO) and indoxyl sulfate (**F**,**G**), liver enzyme (alanine transaminase) (**H**), and serum cytokines (TNF-α, IL-6, and IL-10) (**I**–**K**) are demonstrated (*n* = 11/group).

**Figure 2:**
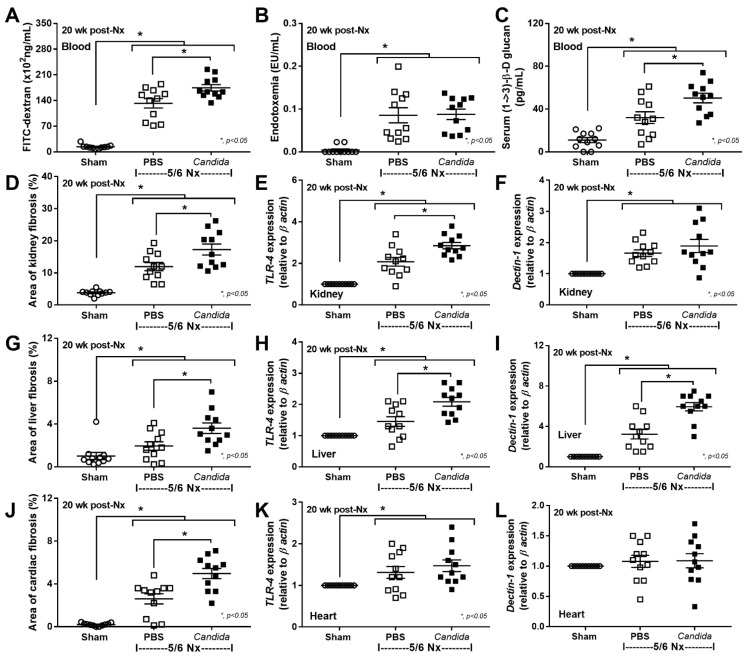
The characteristics of Sham control and 5/6 nephrectomized (5/6 Nx) mice with phosphate buffer solution (PBS) or *Candida* administration (*Candida*) at 20 weeks (wk) after right nephrectomy (20 week post Nx) (see method), as indicated by the gut barrier defect; FITC-dextran assay, endotoxemia, and serum (1→3)-β-D-glucan (BG) (**A**–**C**), area of organ fibrosis with the gene expression of Toll-like receptor 4 (TLR-4) and Dectin-1 in the kidney (**D**–**F**), liver (**G**–**I**), and heart (**J**–**L**) are demonstrated (*n* = 11/group).

**Figure 3:**
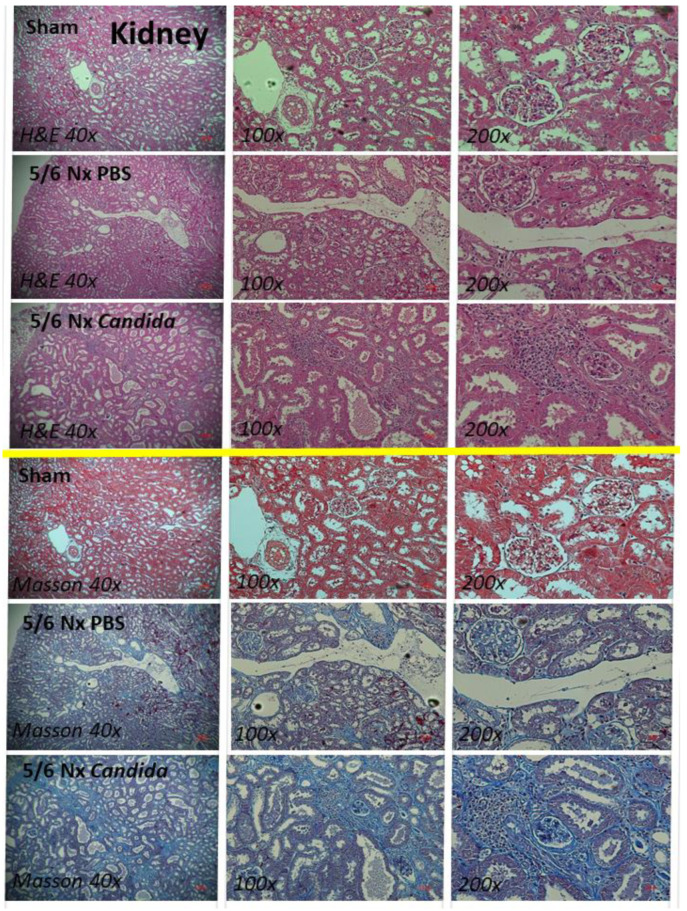
The representative kidney histopathological pictures stained by hematoxylin and eosin (H&E) (**upper part**) or Masson’s trichrome color (**lower part**) (original magnification 40–200×) of Sham control, 5/6 nephrectomized mice with phosphate buffer solution (5/6 Nx PBS) or *Candida* administration (5/6 Nx *Candida*) are demonstrated.

**Figure 4:**
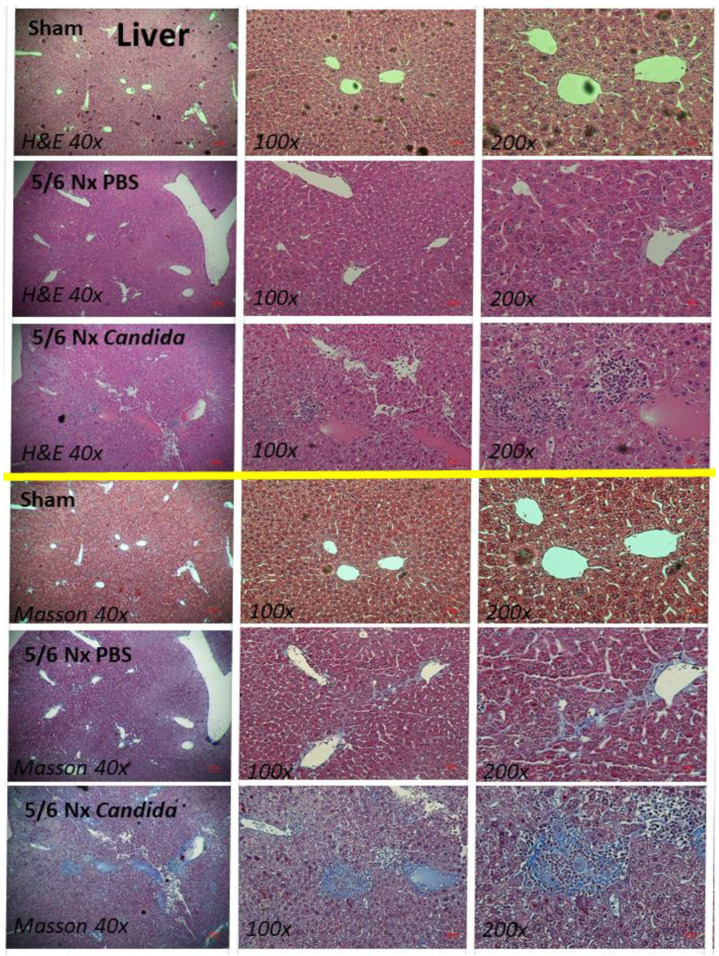
The representative liver histopathological pictures stained by hematoxylin and eosin (H&E) (**upper part**) or Masson’s trichrome color (**lower part**) (original magnification 40–200×) of Sham control, 5/6 nephrectomized mice with phosphate buffer solution (5/6 Nx PBS) or *Candida* administration (5/6 Nx *Candida*) are demonstrated.

**Figure 5:**
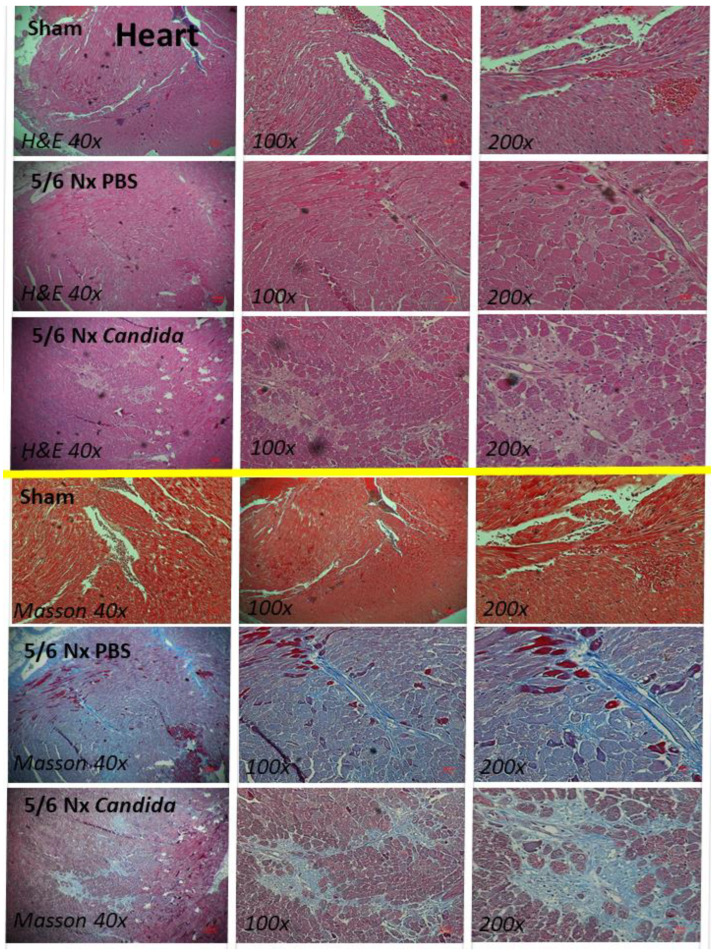
The representative heart histopathological pictures stained by hematoxylin and eosin (H&E) (**upper part**) or Masson’s trichrome color (**lower part**) (original magnification 40–200×) of Sham control, 5/6 nephrectomized mice with phosphate buffer solution (5/6 Nx PBS) or *Candida* administration (5/6 Nx *Candida*) are demonstrated.

Despite the lack of mortality among 5/6 Nx mice regardless of *Candida* administration during the 20 weeks observation, more prominent systemic inflammation and organ injuries in *Candida*-administered 5/6 Nx mice, compared with 5/6 Nx alone, were demonstrated by the increased 24 h proteinuria (a more sensitive renal injury biomarker than serum creatinine) [44], liver enzymes (alanine transaminase), and serum cytokines (TNF-α, IL-6, and IL-10), but not weight loss, systolic blood pressure, hematocrit, serum creatinine, or gut-derived uremic toxins (TMAO and indoxyl sulfate) (Figure 1A–K). The enhanced systemic inflammation might partially be due to the more severe leaky gut in *Candida*-administered 5/6 Nx mice than in 5/6 Nx alone, as indicated by FITC-dextran assay, endotoxemia, and increased serum BG (Figure 2A–C). Notably, the molecular weight (MW) of FITC-dextran is 4.4 kDa, while the MWs of LPS and BG were higher than 0.5–5 kDa, which normally cannot pass through the enterocyte tight junction [10]. In parallel, the increased presentation of LPS and BG, the pathogen-associated molecular patterns (PAMPs) with foreignness properties against the host cells [10], upregulated gene expression of TLR-4 and Dectin-1, the pattern recognition receptor of LPS and BG, respectively, in kidney, liver, and heart, along with the enhanced fibrosis in these organs (Figure 2D–L and Figure 3, Figure 4 and Figure 5), supporting systemic inflammation-induced injury in the kidney, liver, and heart [45,46,47].

### 2.2. An Additive Effect of Lipopolysaccharide (LPS) and (BG) on Inflammation-Induced Fibrosis

**Figure 6:**
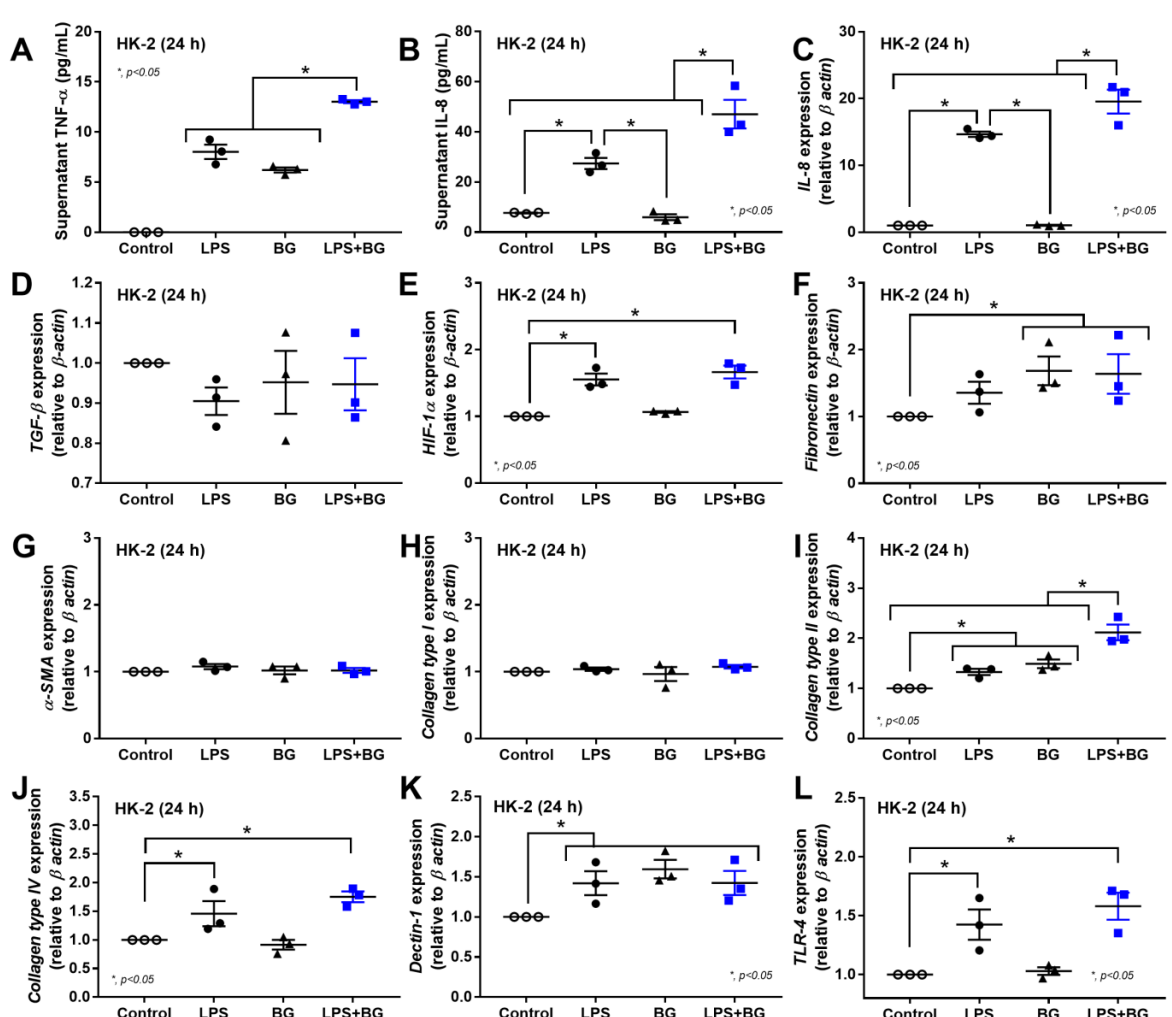
The characteristics of renal tubular cells (HK-2 cells) after 24 h incubation in the culture medium (Control) or lipopolysaccharide (LPS) with or without (1→3)-B-D-glucan (BG), as indicated by supernatant cytokines (TNF-α and IL-8) (**A**,**B**), and the gene expression of cytokines (TGF-β and IL-8) (**C**,**D**), hypoxia-inducible factor-1α (HIF-1α) (**E**), fibrosis-associated genes, including fibronectin, alpha-smooth muscle actin (α-SMA), collagen (type I, III, and IV) (**F**–**J**), and inflammatory mediators (Dectin-1 and Toll-like-receptor 4; TLR-4) (**K**,**L**) are demonstrated. Independent triplicate experiments were performed for all in vitro experiments.

Although inflammatory maladaptation from uremia and LPS alone can cause fibrosis and organ dysfunction [6,48,49], an additive pro-inflammatory impact of BG in fibrosis might also be important. Indeed, LPS plus BG (LPS + BG) induced the highest supernatant IL-6 and TNF-α, with more prominent upregulation of the IL-8 gene when compared with LPS or BG alone in renal tubular cells (Figure 6A–C). Additionally, the enhanced fibrosis after LPS-induced inflammation was demonstrated through the upregulated HIF-1α with collagen type III and IV, but not TGF-β, fibronectin-1, α-SMA, or collagen type I, in renal tubular cells (HK2) (Figure 6D–J). However, the additive effect of BG on LPS-induced fibrosis was indicated only by the upregulation of collagen type III, but not by other genes in these cells (Figure 6D–J). There were subtle responses against BG alone only in supernatant TNF-α and upregulated fibronectin and collagen type III without other parameters in HK2 cells (Figure 6A–J). Interestingly, there was an upregulation of Dectin-1 (a receptor for BG) after induction by either LPS or BG, while TLR-4 was upregulated only with LPS and LPS + BG stimulation (Figure 6K,L), implying a possible LPS–BG synergy through the enhanced BG responses from the LPS-induced Dectin-1 upregulation in renal tubular cells.

**Figure 7:**
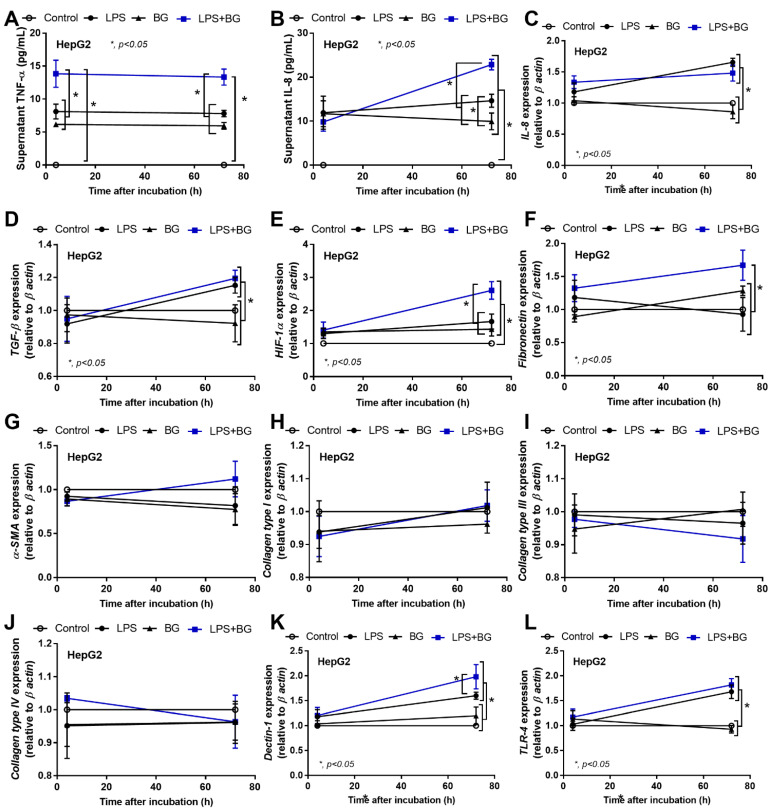
The characteristics of hepatocyte (HepG2 cells) after 1 and 72 h incubation in the culture medium (Control) or lipopolysaccharide (LPS) with or without (1→3)-B-D-glucan (BG), as indicated by supernatant cytokines (TNF-α and IL-8) (**A**,**B**), and the gene expression of cytokines (TGF-β and IL-8) (**C**,**D**), hypoxia-inducible factor-1α (HIF-1α) (**E**), fibrosis-associated genes, including fibronectin, alpha-smooth muscle actin (α-SMA), collagen (type I, III and IV) (**F**–**J**), and inflammatory mediators (Dectin-1 and Toll-like-receptor 4; TLR-4) (**K**,**L**) are demonstrated. Independent triplicate experiments were performed for all in vitro experiments.

Likewise, LPS and BG also enhance inflammation in hepatocytes because LPS and BG from gut translocation are directly transported to the liver through the portal vein [10]. Here, at 1 h after stimulation, supernatant TNF-α, but not other parameters, was elevated, which was more prominent in LPS + BG than the activation by each molecule alone in hepatocytes (HepG2 cells) (Figure 7A–L). Notably, the level of supernatant TNF-α after LPS and BG stimulation was similar (Figure 7A). Then, at 72 h post stimulation, LPS + BG demonstrated the most prominent levels of supernatant IL-8, HIF-1α, and fibronectin, while the expressions of IL-8, TGF-β, Dectin-1, and TLR-4 were similar to LPS stimulation. Interestingly, BG alone obviously increased supernatant cytokines (TNF-α and IL-8) and HIF-1α without an elevation in other parameters in hepatocytes (Figure 7A–L), while it only subtly elevated TNF-α (with Dectin-1) (Figure 7A,K), which is different from the influence of LPS and BG in renal tubular cells (Figure 6K,L), suggesting the different impact of BG on various organs. Nevertheless, our data indicate pro-inflammation-induced fibrosis, especially after LPS stimulation, and the possible LPS–BG additive effects, with less impact via activation with BG alone, on pro-fibrosis in kidney and liver tissue.

## 3. Discussion

### 3.1. Gut Candida Enhanced Systemic Inflammation and Organ Fibrosis in Chronic Kidney Disease (CKD) of 5/6 Nx Mice through Gut Translocation of Glucans

Oral *Candida* administration in mice is used to examine the influence of gut fungi in several models, taking advantage of the lower abundance of *C. albicans* in mouse feces compared with humans (*Candida* in mouse feces is detectable only by polymerase chain reaction (PCR) [50], but not by culture methods [11], which differs from human conditions [12]. Here, *Candida*-5/6 Nx mice had more severe gut leakage (FITC-dextran assay), with a higher level of serum endotoxin and BG (glucanemia) compared to non-*Candida* 5/6 Nx mice, perhaps due to the direct intestinal invasion of fungi, gut dysbiosis, and pro-inflammatory enterocytes [18]. Although enterocytes are naturally resistant to pathogen molecules (LPS and BG), the presence of certain uremic toxins enhances the inflammatory responses in Caco-2 cells (an enterocyte cell line), which might cause a leaky gut [42]. Despite *Candida*-induced gut dysbiosis in uremic mice, as previously described [42], only serum BG, but not gut-derived uremic toxins and endotoxemia, in *Candida* 5/6 Nx were higher than non-*Candida* 5/6 Nx, and serum BG might be the main factor responsible for the hyper-inflammation in *Candida* uremic mice. Additionally, the inflammatory activation of LPS and BG in the blood of 5/6 Nx mice might be responsible for the increased proteinuria, elevated liver enzyme, and organ damage (liver, kidney, and heart), as indicated by histology. Although *Candida* did not alter systolic blood pressure in 5/6 Nx mice, cardiac fibrosis was more prominent in *Candida*-5/6 Nx than 5/6 Nx alone, implying an impact of some non-blood pressure-related profibrotic factors possibly, which was due to the direct myocardial activation by LPS and BG. An upregulation of *TLR-4* and *Dectin-1* in these organs (heart, liver, and kidney) supported an inflammatory synergy of LPS and BG in *Candida*-5/6 Nx mice. Likewise, enhanced fibrosis in the liver might be correlated with LPS–BG from a leaky gut that was directly transported to the liver through the portal vein [51,52]. Notably, the duration of *Candida* administration is important as renal fibrosis at 20 week, but not at 16 weeks of the experiment [18], demonstrated higher fibrosis than the 5/6 Nx control. The enhanced level of BG in serum after *Candida* administration (both heat-killed and viable cells) was also previously demonstrated [31]. It is interesting to note that persistent chronic and severe acute inflammation can frequently lead to maladaptation with organ fibrosis instead of the regular healing process [53]. Here, the chronic inflammation from LPS and BG in serum for 20 weeks is possibly potent enough to generate fibrosis in several internal organs in *Candida*-5/6 Nx mice, supporting the importance of gut fungi in the condition with systemically chronic inflammation. Thus, pro-fibrosis of the internal organs in other conditions with chronic inflammation from leaky gut, such as obesity, inflammatory bowel disease, and cirrhosis, would be interesting to explore further.

### 3.2. The Additive Inflammatory Effect of LPS Plus BG, a Key Pro-Inflammatory Factor in Leaky Gut

The synergy or additive effect of BG presentation upon LPS responses is intensively demonstrated through the activation of several innate immune cells (neutrophils, macrophages, and dendritic cells) [5,17,30,54,55,56,57,58,59,60,61], possibly with the crosslink between TLR-4 and Dectin-1, the pattern recognition receptors (PRRs) for LPS and BG, respectively [62]. The similar downstream signaling through the NF-κB transcription factor of TLR-4 and Dectin-1 in these immune cells may be another explanation of the LPS–BG additive impact on several organs through the activation of immune cells (especially macrophages) inside these organs. Interestingly, trained immunity (enhanced immune response to a second unrelated challenge) through preconditioning BG administration before LPS activation, which is more profound than LPS activation without preconditioning, is also referred to as the LPS–BG synergistic pro-inflammatory effect [63]. However, TLR-4 and Dectin-1 are not only present in immune cells, but also in other parenchymal cells (kidney, liver, and heart) [64,65,66,67,68], which are additively associated with profound inflammation and fibrosis in these organs [34,69,70,71]. Here, the additive inflammation in LPS–BG in hepatocytes might be due to the upregulation of *Dectin-1* by LPS, possibly responsible for the higher responses against BG when simultaneously presented with LPS. The LPS–BG responses of hepatocytes were severe enough for the more profound upregulation of *HIF-1α* and *fibronectin*, the important liver profibrotic genes [72,73], when compared with LPS activation alone. On the other hand, the additive effect of LPS–BG over LPS alone in renal tubular cells was different from the hepatocytes because LPS did not upregulate hepatic *Dectin-1,* but the inflammatory responses against LPS–BG were potent enough for a more prominent *collagen type III* upregulation compared with LPS activation alone [74]. Hence, the underlying mechanisms of the additive LPS–BG pro-inflammatory effect might differ among various organs. Nevertheless, our in vitro data support a possible impact of gut fungi in uremia, partly through the translocation of BG from the gut into the blood circulation, which sub-sequentially facilitated LPS-induced systemic inflammation, possibly through various mechanisms.

### 3.3. Clinical Aspect and Future Experiments

**Figure 8:**
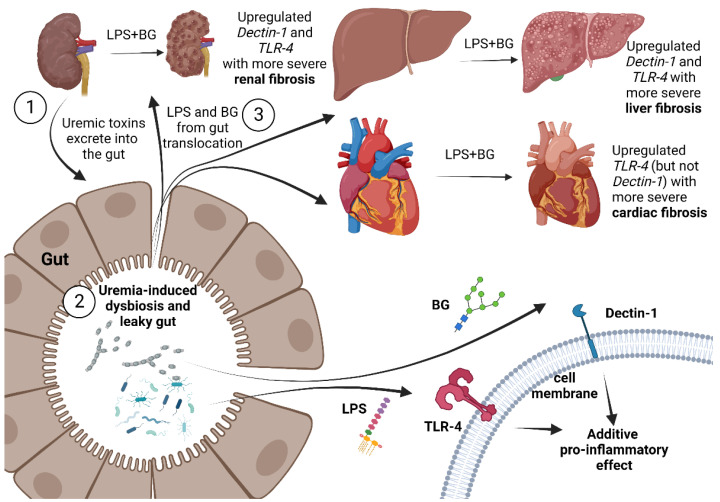
The proposed working hypothesis of the enhanced fibrosis from chronic kidney disease through leaky-gut-induced pro-inflammation that is activated by lipopolysaccharide (LPS) together with (1→3)-β-D-glucan (BG). Firstly (1), the increased uremic toxins in the intestine, and the substitution of kidney excretion, affects gut organisms and causes the intestinal barrier defect. Secondly (2), the LPS and BG from the gut translocate into the blood (leaky gut). Thirdly (3), LPS and BG additively activate pro-inflammation in several organs, partly through the activation of Toll-like-receptor 4 (TLR-4; LPS receptor) and Dectin-1 (BG receptor). The enhanced inflammatory responses from the leaky gut during uremia possibly worsen several uremic complications. The diagram was created using Biorender.com.

Our data support the crosstalk among several organs in CKD through leaky gut, which might be a common situation in several medical conditions (Figure 8). Therefore, measurement of the abundance of LPS and BG in gut content, perhaps through PCR or culture, might be an interesting factor in predicting the severity of systemic inflammation during leaky gut. Additionally, our results support the importance of gut fungi in uremic conditions, especially in patients with severe uremia, before performing kidney replacement therapies (dialysis and renal transplantation). In patients with CKD with positive residual renal function (still producing urine), the determination of leaky gut using an oral administration of non-absorbable carbohydrate before detection in the urine [15,75] might be an interesting biomarker that demonstrates a possibly persistent chronic inflammation from uremia-induced leaky gut. The interventions for the attenuation of leaky gut, such as probiotics and other renal replacement therapies, act through the reduction in uremic toxins (gut-derived and non-gut-derived toxins). The earlier replacement therapies in patients with constant uremia-induced leaky gut might avoid unnecessary leaky-gut-induced hyperinflammation that will lead to injury and fibrosis in several internal organs. Further studies on these topics are warranted for an adaptation of leaky gut in real clinical practice.

## 4. Materials and Methods

### 4.1. Animals and Animal Model

The approved protocol of the Institutional Animal Care and Use Committee of the Faculty of Medicine, Chulalongkorn University, Bangkok, Thailand (CU-ACUP No. 018/2562), according to the National Institutes of Health (NIH) criteria [76], using 8-week-old male C57BL/6 mice, purchased from Nomura Siam (Pathumwan, Bangkok, Thailand), was followed.

#### 4.1.1. *Candida*-Administered Chronic Kidney Disease Model

First, 5/6 nephrectomy (5/6 Nx) surgery was performed via flank approach under isoflurane anesthesia by removing the upper and lower poles of the left kidney before the right nephrectomy 1 week later, as previously described [18,42]. Only mice with a weight of the removed fragments from the left kidney to the right kidney weight in a ratio of 0.55–0.72 were included to ascertain that removal of the left kidney mass is optimum for CKD development [77]. After that, the 5/6 Nx mice were orally administered phosphate buffer solution (PBS) (5/6 Nx PBS) or *Candida albicans* (5/6 Nx + *Candida*) to explore the impact of gut fungi using *C. albicans* from the American Type Culture Collection (ATCC 90028) (Fisher Scientific, Waltham, MA, USA), which was cultured overnight on Sabouraud dextrose broth (SDB) (Oxoid, Hampshire, UK) at 35 °C for 48 h before enumeration using a hemocytometer. The *C. albicans* at 1 × 10^6^ CFU in 0.5 mL PBS or PBS alone was orally administered on alternate days starting from 4 weeks after the right nephrectomy until 20 weeks. Another group of mice underwent the sham operation to identify renal vessels before abdominal closure (Sham group). The *Candida*-administered sham mice have not been demonstrated here because of the lack of difference from the sham control reported in a previous publication [5].

#### 4.1.2. Mouse Sample Analysis

All mice were sacrificed at 20 weeks post right nephrectomy, and the 24 h urine samples were collected at 48 h before sacrifice using a metabolic cage (Hatteras Instruments, Cary, NC, USA). The mouse systolic blood pressure and hematocrit were measured by tail-cuff plethysmography (IITC Life Scientific Instruments, Woodland Hills, CA, USA) [78] and the microhematocrit method with a Coulter counter (Hitachi 917; Boehringer Mannheim GmbH, Mannheim, Germany), respectively. Meanwhile, serum creatinine and 24 h albuminuria were measured by colorimetric method (QuantiChrom™ Creatinine Assay Kit, BioAssay System, Hayward, CA, USA) and enzyme-linked immunosorbent assay (ELISA) (Albuwell M, Exocell™, Philadelphia, PA, USA), respectively. For gut-derived uremic toxins, serum TMAO and indoxyl sulfate were determined by liquid chromatography–mass spectrometry (LC-MS/MS) using a silica column (Luna^®^ silica; 00G-4274-E0, Phenomenex^®^, Torrance, CA, USA) and high-performance liquid chromatography (HPLC Alliance^®^ 2695; Waters, Zellik, Belgium), as previously described [79]. Serum cytokines (TNF-α, IL-6, and IL-10) and liver injury were evaluated by ELISA (Invitrogen, Carlsbad, CA, USA) and EnzyChrom Alanine Transaminase assay (EALT-100, BioAssay, Hayward, CA, USA), respectively. For histopathological analysis, the organs (kidney, liver, and heart) were fixed in 10% formalin, paraffin-embedded, and stained with Masson’s trichrome and hematoxylin and eosin (H&E) colors [38,80]. The area of renal fibrosis in Masson’s-trichrome-stained sections was determined by the computerized image analysis software (ImageJ^©^ software, Bethesda, MD, USA) in a 200× magnification field with 10 fields per sample. Because of the interest in the pro-inflammatory effect of LPS and BG in each organ, the gene expression of *TLR-4* and *Dectin-1* in each organ was evaluated by quantitative reverse transcription polymerase chain reaction (qRT-PCR) relative to *β-actin* (a house-keeping gene) with the 2^−ΔΔCT^ method, as previously described [14,81,82,83]. Briefly, total RNA was extracted from the organs using TRIzol reagent (Invitrogen, Carlsbad, CA, USA) before the conversion into the complementary DNA (cDNA), using 50 ng or the RNA, by high-capacity reverse transcription assay (Applied Biosystems, Warrington, UK) and SYBR Green PCR Master Mix using a QuantStudio™ design and analysis software v1.4.3 (Thermo Fisher Scientific, Foster City, CA, USA) with the rodent primers (Table 1).

#### 4.1.3. Gut Permeability Determination

Because the detection of fluorescein isothiocyanate (FITC)-dextran (an intestinal nonabsorbable carbohydrate) in serum after an oral administration, or the spontaneous elevation in serum of LPS or BG without systemic infection, indicate gut permeability defects (gut leakage), these parameters were used as previously described [30,58,84,85]. As such, 12.5 mg of FITC-dextran (4.4 kDa) (FD4; Sigma-Aldrich^®^, St. Louis, MO, USA) was orally administered before measuring FITC-dextran in serum 3 h later by fluorospectrometer (NanoDrop™ 3300; Thermo Fisher Scientific™, Wilmington, DE, USA). Meanwhile, serum LPS and BG was evaluated by HEK-Blue LPS Detection Kit 2 (InvivoGen™, San Diego, CA, USA) and Fungitell^®^ assay (Associates of Cape Cod, Falmouth, MA, USA), respectively, and values less than 0.01 EU/mL (for LPS) and 7.8 pg/mL (for BG) were recorded as 0, due to the lower limit of the standard curve of the test.

### 4.2. The In Vitro Experiments

To explore a possible pro-fibrotic effect of LPS and BG (a major cell wall component of Gram-negative bacteria and *Candida*, respectively, on kidneys and liver, HK2 renal proximal tubular cells (ATCC 237 CRL-2190) and HepG2 hepatocytes (American Type Culture Collection, Manassas, VA, USA) were used as the representatives. Briefly, HK2 cells or HepG2 at 1 × 10^6^ cells/well maintained in Dulbecco’s modified Eagle Medium (DMEM) were incubated LPS (Escherichia coli O26:B6) (Sigma-Aldrich) at 100 µg/mL with or without BG (CM-Pachyman) (Megazyme, Bray, Ireland) at 100 µg/mL for 24 h (HK2 cells) or 4 and 72 h (HepG2 cells) under 5% CO_2_ at 37 °C before the determination of supernatant cytokines (TNF-α and IL-6) by ELISA (Invitrogen, Carlsbad, CA, USA). Additionally, the expression of several genes related to inflammatory responses and fibrosis was examined by quantitative reverse transcription polymerase chain reaction (qRT-PCR) relative to *β-actin* (a house-keeping gene) with the 2^−ΔΔCT^ method using cDNA (SuperScript™ Vilo™ cDNA synthesis assay) (Invitrogen™) prepared from 50 ng of TRIzol-extracted total RNA (invitrogen™) by a qPCR machine (LightCycler^®^ 2.0) (Roche Diagnostics) with the primers (Table 1).

### 4.3. Statistical Analysis

Analyzed data are presented as mean ± standard error (SE), determined using GraphPad Prism version 9.0 software (La Jolla, CA, USA). Statistical significance was determined by one-way analysis of variance (ANOVA) followed by Tukey’s analysis. The time-point experiments were analyzed by repeated measures ANOVA. All statistical analyses were performed with Stata 16.0 software (StataCorp, TX, USA) and Graph Pad Prism version 7.0 software (La Jolla, CA, USA). A *p*-value of <0.05 was considered statistically significant.

## 5. Conclusions

Our results suggest the crosstalk among several organs in CKD through a leaky gut. Uremia-induced leaky gut in advanced CKD causes the translocation of LPS and BG from the gut into the systemic circulation. As such, LPS and BG additively induce the pro-inflammatory and pro-fibrotic pathways through the activation of TLR-4 and Dectin-1, causing internal organ fibrosis. These enhanced inflammatory responses during leaky gut could worsen uremic complications in patients with advanced CKD. Gut abundance of Gram-negative bacteria and fungi and/or the levels of LPS and BG in the blood might be interesting factors in predicting the severity of systemic inflammation during CKD-induced leaky gut.

## Figures and Tables

**Table 1:** List of the primers.

Name	Forward Primer	Reverse Primer
α-Smooth muscle actin *(α-SMA)* (human)	5′-ACTGAGCGTGGCTATTCCTCCGTT-3′	5′-GCAGTGGCCATCTCATTTTCA-3′
Collagen type I *(Col I)* (human)	5′-CGATGGATTCCAGTTCGAGT-3′	5′-TTTTGAGGGGGTTCAGTTTG-3′
Collagen type III *(Col III)* (human)	5′-GTCCTATTGGTCCTCCTGGC-3′	5′-ACCAGGGAAACCAGCAGG-3′
Collagen type IV *(Col IV)* (human)	5′-ATGGGGCCCCGGCTCAGC-3′	5′-ATCCTCTTTCACCTTTCAATAGC-3′
Dectin-1 (human)	5′-CGCCTCATTGCTGTAATTTTGG-3′	5′-ATCCATCCTCCCAGAGCCA-3′
Fibronectin I (human)	5′-CCGTGGGCAACTCTGTC-3′	5′-TGCGGCAGTTGTCACAG-3′
Hypoxia-inducible factor *(HIF-1α)* (human)	5′-TTCACCTGAGCCTAATAGTCC-3′	5′-CAAGTCTAAATCTGTGTCCTG-3′
Interleukin-8 *(IL-8)* (human)	5′-GAGAGTGATTGAGAGTGGACCAC-3′	5′-CACAACCCTCTGCACCCAGTTT-3′
Toll-like receptor-4 *(TLR-4)* (human)	5′-CACAGACTTGCGGGTTCTAC-3′	5′-AGGACCGACACACCAATGATG -3′
Transforming growth factor β *(TGF-β)* (human)	5′-CAGAGCTGCGCTTGCAGAG-3′	5′-GTCAGCAGCCGGTTACCAAG-3′
β-actin (human)	5′-CCTGGCACCCAGCACAAT-3′	5′-GCCGATCCACACGGAGTACT-3′

## Data Availability

Not applicable.

## References

[1] GBD Chronic Kidney Disease Collaboration (2020). Global, regional, and national burden of chronic kidney disease, 1990-2017: A systematic analysis for the Global Burden of Disease Study 2017. Lancet.

[2] Tang W.H., Wang Z., Levison B.S., Koeth R.A., Britt E.B., Fu X., Wu Y., Hazen S.L. (2013). Intestinal microbial metabolism of phosphatidylcholine and cardiovascular risk. N. Engl. J. Med..

[3] Aronov P.A., Luo F.J., Plummer N.S., Quan Z., Holmes S., Hostetter T.H., Meyer T.W. (2011). Colonic contribution to uremic solutes. J. Am. Soc. Nephrol..

[4] Vaziri N.D., Wong J., Pahl M., Piceno Y.M., Yuan J., DeSantis T.Z., Ni Z., Nguyen T.H., Andersen G.L. (2013). Chronic kidney disease alters intestinal microbial flora. Kidney Int..

[5] Panpetch W., Kullapanich C., Dang C.P., Visitchanakun P., Saisorn W., Wongphoom J., Wannigama D.L., Thim-Uam A., Patarakul K., Somboonna N. (2021). Candida Administration Worsens Uremia-Induced Gut Leakage in Bilateral Nephrectomy Mice, an Impact of Gut Fungi and Organismal Molecules in Uremia. Msystems.

[6] McIntyre C.W., Harrison L.E., Eldehni M.T., Jefferies H.J., Szeto C.C., John S.G., Sigrist M.K., Burton J.O., Hothi D., Korsheed S. (2011). Circulating endotoxemia: A novel factor in systemic inflammation and cardiovascular disease in chronic kidney disease. Clin. J. Am. Soc. Nephrol..

[7] Ellis R.J., Small D.M., Ng K.L., Vesey D.A., Vitetta L., Francis R.S., Gobe G.C., Morais C. (2018). Indoxyl Sulfate Induces Apoptosis and Hypertrophy in Human Kidney Proximal Tubular Cells. Toxicol. Pathol..

[8] Huang Y., Zhou J., Wang S., Xiong J., Chen Y., Liu Y., Xiao T., Li Y., He T., Li Y. (2020). Indoxyl sulfate induces intestinal barrier injury through IRF1-DRP1 axis-mediated mitophagy impairment. Theranostics.

[9] Evenepoel P., Poesen R., Meijers B. (2017). The gut-kidney axis. Pediatr. Nephrol..

[10] Amornphimoltham P., Yuen P.S.T., Star R.A., Leelahavanichkul A. (2019). Gut Leakage of Fungal-Derived Inflammatory Mediators: Part of a Gut-Liver-Kidney Axis in Bacterial Sepsis. Dig. Dis. Sci..

[11] Koh A.Y. (2013). Murine models of Candida gastrointestinal colonization and dissemination. Eukaryot. Cell.

[12] Borges F.M., de Paula T.O., Sarmiento M.R.A., de Oliveira M.G., Pereira M.L.M., Toledo I.V., Nascimento T.C., Ferreira-Machado A.B., Silva V.L., Diniz C.G. (2018). Fungal Diversity of Human Gut Microbiota Among Eutrophic, Overweight, and Obese Individuals Based on Aerobic Culture-Dependent Approach. Curr. Microbiol..

[13] Iliev I.D., Leonardi I. (2017). Fungal dysbiosis: Immunity and interactions at mucosal barriers. Nat. Rev. Immunol..

[14] Panpetch W., Phuengmaung P., Hiengrach P., Issara-Amphorn J., Cheibchalard T., Somboonna N., Tumwasorn S., Leelahavanichkul A. (2022). Candida Worsens Klebsiella pneumoniae Induced-Sepsis in a Mouse Model with Low Dose Dextran Sulfate Solution through Gut Dysbiosis and Enhanced Inflammation. Int. J. Mol. Sci..

[15] Chancharoenthana W., Kamolratanakul S., Ariyanon W., Thanachartwet V., Phumratanaprapin W., Wilairatana P., Leelahavanichkul A. (2022). Abnormal Blood Bacteriome, Gut Dysbiosis, and Progression to Severe Dengue Disease. Front. Cell. Infect. Microbiol..

[16] Hiengrach P., Panpetch W., Chindamporn A., Leelahavanichkul A. (2022). Macrophage depletion alters bacterial gut microbiota partly through fungal overgrowth in feces that worsens cecal ligation and puncture sepsis mice. Sci. Rep..

[17] Saithong S., Worasilchai N., Saisorn W., Udompornpitak K., Bhunyakarnjanarat T., Chindamporn A., Tovichayathamrong P., Torvorapanit P., Chiewchengchol D., Chancharoenthana W. (2022). Neutrophil Extracellular Traps in Severe SARS-CoV-2 Infection: A Possible Impact of LPS and (1→3)-beta-D-glucan in Blood from Gut Translocation. Cells.

[18] Tungsanga S., Panpetch W., Bhunyakarnjanarat T., Udompornpitak K., Katavetin P., Chancharoenthana W., Chatthanathon P., Somboonna N., Tungsanga K., Tumwasorn S. (2022). Uremia-Induced Gut Barrier Defect in 5/6 Nephrectomized Mice Is Worsened by Candida Administration through a Synergy of Uremic Toxin, Lipopolysaccharide, and (13)-beta-D-Glucan, but Is Attenuated by Lacticaseibacillus rhamnosus L34. Int. J. Mol. Sci..

[19] Thim-Uam A., Makjaroen J., Issara-Amphorn J., Saisorn W., Wannigama D.L., Chancharoenthana W., Leelahavanichkul A. (2022). Enhanced Bacteremia in Dextran Sulfate-Induced Colitis in Splenectomy Mice Correlates with Gut Dysbiosis and LPS Tolerance. Int. J. Mol. Sci..

[20] Kaewduangduen W., Visitchanakun P., Saisorn W., Phawadee A., Manonitnantawat C., Chutimaskul C., Susantitaphong P., Ritprajak P., Somboonna N., Cheibchalard T. (2022). Blood Bacteria-Free DNA in Septic Mice Enhances LPS-Induced Inflammation in Mice through Macrophage Response. Int. J. Mol. Sci..

[21] Liu G.L., Chi Z., Wang G.Y., Wang Z.P., Li Y., Chi Z.M. (2015). Yeast killer toxins, molecular mechanisms of their action and their applications. Crit. Rev. Biotechnol..

[22] Chu H., Duan Y., Lang S., Jiang L., Wang Y., Llorente C., Liu J., Mogavero S., Bosques-Padilla F., Abraldes J.G. (2020). The Candida albicans exotoxin candidalysin promotes alcohol-associated liver disease. J. Hepatol..

[23] Geertinger P., Bodenhoff J., Helweg-Larsen K., Lund A. (1982). Endogenous alcohol production by intestinal fermentation in sudden infant death. Z. Rechtsmed..

[24] Kaji H., Asanuma Y., Yahara O., Shibue H., Hisamura M., Saito N., Kawakami Y., Murao M. (1984). Intragastrointestinal alcohol fermentation syndrome: Report of two cases and review of the literature. J. Forensic. Sci. Soc..

[25] Bauer M.A., Kainz K., Carmona-Gutierrez D., Madeo F. (2018). Microbial wars: Competition in ecological niches and within the microbiome. Microb. Cell.

[26] Hiengrach P., Panpetch W., Worasilchai N., Chindamporn A., Tumwasorn S., Jaroonwitchawan T., Wilantho A., Chatthanathon P., Somboonna N., Leelahavanichkul A. (2020). Administration of Candida Albicans to Dextran Sulfate Solution Treated Mice Causes Intestinal Dysbiosis, Emergence and Dissemination of Intestinal Pseudomonas Aeruginosa and Lethal Sepsis. Shock.

[27] Charoensappakit A., Sae-Khow K., Leelahavanichkul A. (2022). Gut Barrier Damage and Gut Translocation of Pathogen Molecules in Lupus, an Impact of Innate Immunity (Macrophages and Neutrophils) in Autoimmune Disease. Int. J. Mol. Sci..

[28] Panpetch W., Somboonna N., Palasuk M., Hiengrach P., Finkelman M., Tumwasorn S., Leelahavanichkul A. (2019). Oral Candida administration in a Clostridium difficile mouse model worsens disease severity but is attenuated by Bifidobacterium. PLoS ONE.

[29] Panpetch W., Hiengrach P., Nilgate S., Tumwasorn S., Somboonna N., Wilantho A., Chatthanathon P., Prueksapanich P., Leelahavanichkul A. (2020). Additional Candida albicans administration enhances the severity of dextran sulfate solution induced colitis mouse model through leaky gut-enhanced systemic inflammation and gut-dysbiosis but attenuated by Lactobacillus rhamnosus L34. Gut Microbes.

[30] Panpetch W., Sawaswong V., Chanchaem P., Ondee T., Dang C.P., Payungporn S., Tumwasorn S., Leelahavanichkul A. (2020). Corrigendum: Candida Administration Worsens Cecal Ligation and Puncture-Induced Sepsis in Obese Mice Through Gut Dysbiosis Enhanced Systemic Inflammation, Impact of Pathogen-Associated Molecules From Gut Translocation and Saturated Fatty Acid. Front. Immunol..

[31] Panpetch W., Somboonna N., Bulan D.E., Issara-Amphorn J., Finkelman M., Worasilchai N., Chindamporn A., Palaga T., Tumwasorn S., Leelahavanichkul A. (2017). Oral administration of live- or heat-killed Candida albicans worsened cecal ligation and puncture sepsis in a murine model possibly due to an increased serum (1→3)-beta-D-glucan. PLoS ONE.

[32] Panpetch W., Somboonna N., Bulan D.E., Issara-Amphorn J., Worasilchai N., Finkelman M., Chindamporn A., Palaga T., Tumwasorn S., Leelahavanichkul A. (2018). Gastrointestinal Colonization of Candida Albicans Increases Serum (1→3)-beta-D-Glucan, without Candidemia, and Worsens Cecal Ligation and Puncture Sepsis in Murine Model. Shock.

[33] Yang A.M., Inamine T., Hochrath K., Chen P., Wang L., Llorente C., Bluemel S., Hartmann P., Xu J., Koyama Y. (2017). Intestinal fungi contribute to development of alcoholic liver disease. J. Clin. Investig..

[34] Seifert L., Deutsch M., Alothman S., Alqunaibit D., Werba G., Pansari M., Pergamo M., Ochi A., Torres-Hernandez A., Levie E. (2015). Dectin-1 Regulates Hepatic Fibrosis and Hepatocarcinogenesis by Suppressing TLR4 Signaling Pathways. Cell. Rep..

[35] Li X., Bian Y., Pang P., Yu S., Wang X., Gao Y., Liu K., Liu Q., Yuan Y., Du W. (2020). Inhibition of Dectin-1 in mice ameliorates cardiac remodeling by suppressing NF-kappaB/NLRP3 signaling after myocardial infarction. Int. Immunopharmacol..

[36] Calabrese D.R., Wang P., Chong T., Hoover J., Singer J.P., Torgerson D., Hays S.R., Golden J.A., Kukreja J., Dugger D. (2019). Dectin-1 genetic deficiency predicts chronic lung allograft dysfunction and death. JCI Insight.

[37] Brown G.D., Herre J., Williams D.L., Willment J.A., Marshall A.S., Gordon S. (2003). Dectin-1 mediates the biological effects of beta-glucans. J. Exp. Med..

[38] Chancharoenthana W., Leelahavanichkul A., Taratummarat S., Wongphom J., Tiranathanagul K., Eiam-Ong S. (2017). Cilostazol attenuates intimal hyperplasia in a mouse model of chronic kidney disease. PLoS ONE.

[39] Vanholder R., Schepers E., Pletinck A., Nagler E.V., Glorieux G. (2014). The uremic toxicity of indoxyl sulfate and p-cresyl sulfate: A systematic review. J. Am. Soc. Nephrol..

[40] Wu I.W., Hsu K.H., Lee C.C., Sun C.Y., Hsu H.J., Tsai C.J., Tzen C.Y., Wang Y.C., Lin C.Y., Wu M.S. (2011). p-Cresyl sulphate and indoxyl sulphate predict progression of chronic kidney disease. Nephrol. Dial. Transpl..

[41] Panpetch W., Chancharoenthana W., Bootdee K., Nilgate S., Finkelman M., Tumwasorn S., Leelahavanichkul A. (2018). Lactobacillus rhamnosus L34 Attenuates Gut Translocation-Induced Bacterial Sepsis in Murine Models of Leaky Gut. Infect. Immun..

[42] Tungsanga S., Katavetin P., Panpetch W., Udompornpitak K., Saisorn W., Praditpornsilpa K., Eiam-Ong S., Tungsanga K., Tumwasorn S., Leelahavanichkul A. (2022). Lactobacillus rhamnosus L34 attenuates chronic kidney disease progression in a 5/6 nephrectomy mouse model through the excretion of anti-inflammatory molecules. Nephrol. Dial. Transpl..

[43] Forkosh E., Ilan Y. (2019). The heart-gut axis: New target for atherosclerosis and congestive heart failure therapy. Open. Heart.

[44] Obert L.A., Elmore S.A., Ennulat D., Frazier K.S. (2021). A Review of Specific Biomarkers of Chronic Renal Injury and Their Potential Application in Nonclinical Safety Assessment Studies. Toxicol. Pathol..

[45] Effenberger M., Grander C., Grabherr F., Griesmacher A., Ploner T., Hartig F., Bellmann-Weiler R., Joannidis M., Zoller H., Weiss G. (2021). Systemic inflammation as fuel for acute liver injury in COVID-19. Dig. Liver. Dis..

[46] Trimarchi H., Muryan A., Dicugno M., Young P., Forrester M., Lombi F., Pomeranz V., Iriarte R., Rana M.S., Alonso M. (2012). Proteinuria: An ignored marker of inflammation and cardiovascular disease in chronic hemodialysis. Int. J. Nephrol. Renovasc. Dis..

[47] Li Y., Ge S., Peng Y., Chen X. (2013). Inflammation and cardiac dysfunction during sepsis, muscular dystrophy, and myocarditis. Burns. Trauma.

[48] Andrade-Oliveira V., Foresto-Neto O., Watanabe I.K.M., Zatz R., Camara N.O.S. (2019). Inflammation in Renal Diseases: New and Old Players. Front. Pharmacol..

[49] Mohamed H.A., Elbastawisy Y.M., Elsaed W.M. (2019). Attenuation of lipopolysaccharide-induced lung inflammation by ascorbic acid in rats: Histopathological and ultrastructural study. SAGE Open Med..

[50] Heisel T., Montassier E., Johnson A., Al-Ghalith G., Lin Y.W., Wei L.N., Knights D., Gale C.A. (2017). High-Fat Diet Changes Fungal Microbiomes and Interkingdom Relationships in the Murine Gut. Msphere.

[51] Wang Y., Zhang Y., Liu Y., Xu J., Liu Y. (2021). Gut-Liver Axis: Liver Sinusoidal Endothelial Cells Function as the Hepatic Barrier in Colitis-Induced Liver Injury. Front. Cell. Dev. Biol..

[52] Prytz H., Holst-Christensen J., Korner B., Liehr H. (1976). Portal venous and systemic endotoxaemia in patients without liver disease and systemic endotoxaemia in patients with cirrhosis. Scand. J. Gastroenterol..

[53] Wynn T.A., Ramalingam T.R. (2012). Mechanisms of fibrosis: Therapeutic translation for fibrotic disease. Nat. Med..

[54] Issara-Amphorn J., Chancharoenthana W., Visitchanakun P., Leelahavanichkul A. (2020). Syk Inhibitor Attenuates Polymicrobial Sepsis in FcgRIIb-Deficient Lupus Mouse Model, the Impact of Lupus Characteristics in Sepsis. J. Innate Immun..

[55] Issara-Amphorn J., Dang C.P., Saisorn W., Limbutara K., Leelahavanichkul A. (2021). Candida Administration in Bilateral Nephrectomy Mice Elevates Serum (1→3)-beta-D-glucan That Enhances Systemic Inflammation Through Energy Augmentation in Macrophages. Int. J. Mol. Sci..

[56] Issara-Amphorn J., Somboonna N., Pisitkun P., Hirankarn N., Leelahavanichkul A. (2020). Syk inhibitor attenuates inflammation in lupus mice from FcgRIIb deficiency but not in pristane induction: The influence of lupus pathogenesis on the therapeutic effect. Lupus.

[57] Issara-Amphorn J., Surawut S., Worasilchai N., Thim-Uam A., Finkelman M., Chindamporn A., Palaga T., Hirankarn N., Pisitkun P., Leelahavanichkul A. (2018). The Synergy of Endotoxin and (1→3)-beta-D-Glucan, from Gut Translocation, Worsens Sepsis Severity in a Lupus Model of Fc Gamma Receptor IIb-Deficient Mice. J. Innate Immun..

[58] Sae-Khow K., Charoensappakit A., Visitchanakun P., Saisorn W., Svasti S., Fucharoen S., Leelahavanichkul A. (2020). Pathogen-Associated Molecules from Gut Translocation Enhance Severity of Cecal Ligation and Puncture Sepsis in Iron-Overload beta-Thalassemia Mice. J. Inflamm. Res..

[59] Saithong S., Saisorn W., Dang C.P., Visitchanakun P., Chiewchengchol D., Leelahavanichkul A. (2022). Candida Administration Worsens Neutrophil Extracellular Traps in Renal Ischemia Reperfusion Injury Mice: An Impact of Gut Fungi on Acute Kidney Injury. J. Innate Immun..

[60] Saithong S., Saisorn W., Visitchanakun P., Sae-Khow K., Chiewchengchol D., Leelahavanichkul A. (2021). A Synergy Between Endotoxin and (1→3)-Beta-D-Glucan Enhanced Neutrophil Extracellular Traps in Candida Administered Dextran Sulfate Solution Induced Colitis in FcGRIIB-/- Lupus Mice, an Impact of Intestinal Fungi in Lupus. J. Inflamm. Res..

[61] Thim-Uam A., Surawut S., Issara-Amphorn J., Jaroonwitchawan T., Hiengrach P., Chatthanathon P., Wilantho A., Somboonna N., Palaga T., Pisitkun P. (2020). Leaky-gut enhanced lupus progression in the Fc gamma receptor-IIb deficient and pristane-induced mouse models of lupus. Sci. Rep..

[62] Ferwerda G., Meyer-Wentrup F., Kullberg B.J., Netea M.G., Adema G.J. (2008). Dectin-1 synergizes with TLR2 and TLR4 for cytokine production in human primary monocytes and macrophages. Cell. Microbiol..

[63] Benjaskulluecha S., Boonmee A., Pattarakankul T., Wongprom B., Klomsing J., Palaga T. (2022). Screening of compounds to identify novel epigenetic regulatory factors that affect innate immune memory in macrophages. Sci. Rep..

[64] Lech M., Susanti H.E., Rommele C., Grobmayr R., Gunthner R., Anders H.J. (2012). Quantitative expression of C-type lectin receptors in humans and mice. Int. J. Mol. Sci..

[65] Kumar S., Wang J., Shanmukhappa S.K., Gandhi C.R. (2017). Toll-Like Receptor 4-Independent Carbon Tetrachloride-Induced Fibrosis and Lipopolysaccharide-Induced Acute Liver Injury in Mice: Role of Hepatic Stellate Cells. Am. J. Pathol..

[66] Liu F., Wen Y., Kang J., Wei C., Wang M., Zheng Z., Peng J. (2018). Regulation of TLR4 expression mediates the attenuating effect of erythropoietin on inflammation and myocardial fibrosis in rat heart. Int. J. Mol. Med..

[67] Skuginna V., Lech M., Allam R., Ryu M., Clauss S., Susanti H.E., Rommele C., Garlanda C., Mantovani A., Anders H.J. (2011). Toll-like receptor signaling and SIGIRR in renal fibrosis upon unilateral ureteral obstruction. PLoS ONE.

[68] Yu L., Feng Z. (2018). The Role of Toll-Like Receptor Signaling in the Progression of Heart Failure. Mediat. Inflamm..

[69] Pradere J.P., Troeger J.S., Dapito D.H., Mencin A.A., Schwabe R.F. (2010). Toll-like receptor 4 and hepatic fibrogenesis. Semin. Liver. Dis..

[70] Pulskens W.P., Rampanelli E., Teske G.J., Butter L.M., Claessen N., Luirink I.K., van der Poll T., Florquin S., Leemans J.C. (2010). TLR4 promotes fibrosis but attenuates tubular damage in progressive renal injury. J. Am. Soc. Nephrol..

[71] Ge C., Zhao Y., Liang Y., He Y. (2022). Silencing of TLR4 Inhibits Atrial Fibrosis and Susceptibility to Atrial Fibrillation via Downregulation of NLRP3-TGF-beta in Spontaneously Hypertensive Rats. Dis. Markers.

[72] Liu X.Y., Liu R.X., Hou F., Cui L.J., Li C.Y., Chi C., Yi E., Wen Y., Yin C.H. (2016). Fibronectin expression is critical for liver fibrogenesis in vivo and in vitro. Mol. Med. Rep..

[73] Han J., He Y., Zhao H., Xu X. (2019). Hypoxia inducible factor-1 promotes liver fibrosis in nonalcoholic fatty liver disease by activating PTEN/p65 signaling pathway. J. Cell. Biochem..

[74] Dong X., Li Y., Cao R., Xu H. (2021). MicroRNA-363-3p Inhibits the Expression of Renal Fibrosis Markers in TGF-beta1-Treated HK-2 Cells by Targeting TGF-beta2. Biochem. Genet..

[75] Chancharoenthana W., Leelahavanichkul A., Ariyanon W., Vadcharavivad S., Phatcharophaswattanakul S., Kamolratanakul S., Leaungwutiwong P., Phumratanaprapin W., Wilairatana P. (2021). Leaky Gut Syndrome Is Associated with Endotoxemia and Serum (1→3)-beta-D-Glucan in Severe Dengue Infection. Microorganisms.

[76] National Research Council (US) (2011). Guide for the Care and Use of Laboratory Animals.

[77] Leelahavanichkul A., Yan Q., Hu X., Eisner C., Huang Y., Chen R., Mizel D., Zhou H., Wright E.C., Kopp J.B. (2010). Angiotensin II overcomes strain-dependent resistance of rapid CKD progression in a new remnant kidney mouse model. Kidney Int..

[78] Seujange Y., Leelahavanichkul A., Yisarakun W., Khawsuk W., Meepool A., Phamonleatmongkol P., Saechau W., Onlamul W., Tantiwarattanatikul P., Oonsook W. (2013). Hibiscus sabdariffa Linnaeus aqueous extracts attenuate the progression of renal injury in 5/6 nephrectomy rats. Ren. Fail..

[79] Boonhai S., Bootdee K., Saisorn W., Takkavatakarn K., Sitticharoenchai P., Tungsanga S., Tiranathanagul K., Leelahavanichkul A. (2021). TMAO reductase, a biomarker for gut permeability defect induced inflammation, in mouse model of chronic kidney disease and dextran sulfate solution-induced mucositis. Asian Pac. J. Allergy Immunol..

[80] Udompornpitak K., Charoensappakit A., Sae-Khow K., Bhunyakarnjanarat T., Dang C.P., Saisorn W., Visitchanakun P., Phuengmaung P., Palaga T., Ritprajak P. (2022). Obesity Exacerbates Lupus Activity in Fc Gamma Receptor IIb Deficient Lupus Mice Partly through Saturated Fatty Acid-Induced Gut Barrier Defect and Systemic Inflammation. J. Innate Immun..

[81] Hiengrach P., Visitchanakun P., Tongchairawewat P., Tangsirisatian P., Jungteerapanich T., Ritprajak P., Wannigama D.L., Tangtanatakul P., Leelahavanichkul A. (2022). Sepsis Encephalopathy Is Partly Mediated by miR370-3p-Induced Mitochondrial Injury but Attenuated by BAM15 in Cecal Ligation and Puncture Sepsis Male Mice. Int. J. Mol. Sci..

[82] Panpetch W., Phuengmaung P., Cheibchalard T., Somboonna N., Leelahavanichkul A., Tumwasorn S. (2021). Lacticaseibacillus casei Strain T21 Attenuates Clostridioides difficile Infection in a Murine Model Through Reduction of Inflammation and Gut Dysbiosis With Decreased Toxin Lethality and Enhanced Mucin Production. Front. Microbiol..

[83] Visitchanakun P., Tangtanatakul P., Trithiphen O., Soonthornchai W., Wongphoom J., Tachaboon S., Srisawat N., Leelahavanichkul A. (2020). Plasma miR-370-3P as a Biomarker of Sepsis-Associated Encephalopathy, the Transcriptomic Profiling Analysis of Microrna-Arrays From Mouse Brains. Shock.

[84] Visitchanakun P., Saisorn W., Wongphoom J., Chatthanathon P., Somboonna N., Svasti S., Fucharoen S., Leelahavanichkul A. (2020). Gut leakage enhances sepsis susceptibility in iron-overloaded beta-thalassemia mice through macrophage hyperinflammatory responses. Am. J. Physiol. Gastrointest. Liver. Physiol..

[85] Bhunyakarnjanarat T., Udompornpitak K., Saisorn W., Chantraprapawat B., Visitchanakun P., Dang C.P., Issara-Amphorn J., Leelahavanichkul A. (2021). Prominent Indomethacin-Induced Enteropathy in Fcgriib Defi-cient lupus Mice: An Impact of Macrophage Responses and Immune Deposition in Gut. Int. J. Mol. Sci..

